# From 2D to 3D gamma passing rate tolerance and action limits for patient‐specific quality assurance in volumetric‐modulated arc therapy

**DOI:** 10.1002/acm2.70025

**Published:** 2025-02-19

**Authors:** Christos Zarros, George Patatoukas, Nikos Kollaros, Marina Chalkia, Andromachi Kougioumtzopoulou, Vasilios Kouloulias, Kalliopi Platoni

**Affiliations:** ^1^ Medical Physics Unit, Second Department of Radiology, Medical School National and Kapodistrian University of Athens Attikon General University Hospital Haidari Athens Greece; ^2^ Radiation Therapy Unit, Second Department of Radiology Medical School National and Kapodistrian University of Athens Attikon General University Hospital Haidari Athens Greece

**Keywords:** action limits (AL), Gamma passing rate (%GP), tolerance Limits (TL), VMAT PSQA

## Abstract

**Background:**

Volumetric modulated arc therapy (VMAT) requires an accurate patient‐specific quality assurance (PSQA) program. In clinical practice, this is usually performed using the γ‐index and the two‐dimensional gamma passing rate (2D %GP). A three‐dimensional (3D) index incorporating the patient anatomy could be more useful for the 3D dose distribution verification.

**Purpose:**

The current study demonstrates a thorough investigation of VMAT PSQA treatment plans by examining the correlation between 3D Gamma passing rate (%GP) and 2D %GP. The aim was to establish the tolerance limits (TL) and action limits (AL) that could be adopted in clinical practice.

**Materials and methods:**

PSQA was performed for 67 head and neck (H&N) and 69 prostate treatment plans, using an appropriate phantom and the γ‐index method. The 3%/2  mm acceptance criterion was used. Treatment plans’ 2D% GP and 3D %GP values were collected and correlated with individual 3D %GP values of planning target volume (PTV) and organs at risk (OARs). Institutional TL and AL of both 2D %GP and 3D %GP were established using 30 prostate and 30 H&N treatment plans, as per recommendations proposed by AAPM TG‐218.

**Results:**

A moderate correlation was observed between 2D %GP and 3D %GP of the treatment plans. Τhe correlations demonstrated a stronger association for the 3D %GP than for the 2D %GP with respect to the 3D %GP of the individual structures considered. The TL for the 3D %GP (both plan and individual) and the plans’ 2D %GP were generally more stringent, while the AL showed a wider range compared to the corresponding limits suggested by the TG‐218 protocol for plan 2D %GP.

**Conclusions:**

Institution‐specific 3D %GP as well as TL and AL for treatment plan, PTV and OARs could be incorporated in the PSQA procedure in synergy with the 2D evaluation, as they can provide a more‐in‐depth‐view of the treatment quality.

## 1 INTRODUCTION

Volumetric modulated arc therapy (VMAT) is a modern radiotherapy technique, which utilizes multi‐leaf collimator (MLC) dynamic motion coupled with gantry angle rotation and dose rate variation, resulting in the beam intensity being continuously varied to irradiate the target volume with increased compliance, accuracy, and beam efficiency. It is a technique of high complexity and thus it requires appropriate quality assurance (QA) of each plan to be performed.^1^

A comprehensive QA program should encompass all areas of the treatment process to ensure that the treatment plan is delivered accurately, especially for complex techniques such as VMAT.^2^ This requires patient‐specific quality assurance (PSQA) measurements, sensitive enough to detect errors and/or failures that may arise in the treatment process, taking into account the desired level of accuracy and the local resources available in the institution and in accordance with national and international guidelines.^3^ This monitoring is performed either by using a suitable phantom or by applying computational methods based on the comparison of log files.^1^

In clinical practice, VMAT PSQA is carried out by comparing the measured dose distribution from the linear accelerator (LINAC) with the calculated dose distribution from the TPS. The comparison is performed through the γ‐index, introduced by Low et al.^4^ More precisely, the γ‐index compares dose points in terms of their percentage dose difference (%DD) and distance to agreement (DTA).^2^ By calculating the γ‐index for every point in the reference dose distribution the percentage of points that have attained *γ* < 1 for a specific criterion is computed. This percentage defines the %GP (Gamma passing rate). In addition, a pass/fail threshold is established.^5^ By definition the %GP should be accompanied by an acceptance criterion in the form of %DD/DTA (mm). Moreover, there is a low‐dose cutoff threshold below which the γ‐index is not calculated to exclude the low‐dose areas from the %GP calculations (off‐field). This cutoff threshold is set to 10% of the plan's maximum dose.^6^, ^7^ AAPM TG‐119 publication recommended that a test is considered successful when more than 90% of the points (γ passing rate, %GP) of the measured plan agree with the calculated ones, using the %DD/DTA criteria of 3%/3 mm.^8^ With the 2018 updated protocol on Tolerance and Action Limits (TL and AL respectively), AAPM TG‐218 recommended changing the criteria to 3%/2  mm and TL as 95% and AL as 90%.^6^

Despite the fact that the two‐dimensional (2D) %GP is a valuable tool for the PSQA of the plans, it is a 2D evaluation index with inherent limitations. Specifically, it does not provide any information regarding the location of the points (both fail and pass) with respect to patient anatomy. Various studies have demonstrated that 2D %GP does not have a strong correlation with clinical dose parameters.^9^, ^10^, ^11^, ^12^, ^13^, ^14^, ^15^, ^16^, ^17^, ^18^ Because of this lack of correlation between phantom and patient‐anatomy dose distribution, a three‐dimensional (3D) index could be useful for the 3D dose verification based on the actual patient anatomy. One of the available commercial software is the 3DVH (Sun Nuclear Corporation, Melbourne, FL, USA). It can provide the %GP and calculate the Dose Volume Histograms (DVHs) for each structure and target volume, using the LINAC‐based measurements.

This study aimed to investigate and compare the delivery verification of VMAT plans using 2D and 3D %GP indexes, within a 3%/2  mm acceptance criterion. Furthermore, correlations between plan 2D %GP and plan 3D %GP, plan 2D %GP and individual 3D %GP of each structure and plan 3D %GP versus 3D %GP (individual) of each structure were explored. The analysis of these correlations could provide useful insight into treatment plans’ level of complexity.

Similar investigations have been carried out in previous studies such as Song et al.^18^ and Dandan Zhang et al.,^19^ who performed correlations between plan 2D %GP and 3D %GP (plan and individual). Further studies^12^, ^13^, ^15^, ^16^, ^17^, ^20^, ^21^, ^22^, ^23^ have investigated %dose errors and other studies have correlated %GP with %DE (%dose errors).^12^, ^16^, ^17^, ^20^, ^21^, ^22^, ^24^

Furthermore, this study attempted to establish TL and AL for both 3D %GP (plan and individual) and plan 2D %GP, following the work of Stasinou et al.,^21^ in which TL and AL of plan 2D %GP were determined. Such an analysis can be considered particularly helpful, because a high 3D or 2D %GP of a treatment plan does not necessarily correlate with a high individual 3D %GP of a structure. Similarly, a low treatment plan %GP does not necessarily indicate a low %GP in a structure. It therefore seems necessary to determine TL and AL for both 3D %GP (plan and individual).

## 2 MATERIALS AND METHODS

### 2.1 Patients

The investigated cohort comprised of 23 patients with head and neck (H&N) malignancies, encompassing a cumulative total of 67 treatment plans, and 30 patients with prostate cancer, involving a cumulative total of 69 treatment plans. The treatment regimens for each patient involved sequential VMAT treatments (sequential boost), escalating the dose according to lower, intermediate and high‐risk planning target volumes (PTVs) through distinct treatment plans.

In prostate cases, the treatment delivery involved two or three successive treatment steps, while H&N cases consisted of three treatment steps. The conventional fractionation scheme of 2 Gy/fraction was followed for all treatment steps. The dose fractionation schemes used were the following:

*Prostate*: Phase 1, 2, 3 → Group A: (46, 56, 76) Gy/Group B: (46, 76) Gy/Group C: (56, 76) Gy.

*H&N*: Phase 1, 2, 3 → (54, 60, 70) Gy.

All VMAT plans were calculated using a 2.5 mm isotropic dose grid with 6 MV photon beam using the TPS Eclipse (v.17.1., Varian Medical Systems) software. The PTV and organs at risk (OARs) were delineated by expert radiation oncologists, and the treatment plans were created, optimized, and calculated by the medical physicists using the anisotropic analytical algorithm (version 17.1).

### 2.2 PSQA

PSQA was performed in VitalBeam linear accelerators (Varian Medical Systems) equipped with the Millennium 120 MLC, using the ArcCHECK phantom (Model 1220, Sun Nuclear Corporation, Melbourne, FL, USA). It is a cylindrical water‐equivalent phantom, containing a 3D array of 1386 n‐type solid state diode detectors, arranged in a helical pattern. The inside of the phantom has a cavity, where various inserts can be accommodated. For this study the CavityPlug was inserted, a solid polymethylmethacrylate (PMMA) core (water equivalent material), in order to allow measurements with homogeneous insert.

The analysis of dose distributions (measured and calculated) in two dimensions was performed with the SNC Patient software (v.6.7.4, Sun Nuclear Corporation). The SNC Patient software calculates the %GP using the γ‐index. Its interface projects the 2D measured dose distribution to the TPS calculated one, in order to compare the two distributions. Through this comparison, the gamma analysis of dose/fluence map is provided. The resulting %GP for the 3%/2  mm criterion with global normalization and 10% of the maximum low‐dose cutoff threshold was recorded and used for the correlations in this study.

### 2.3 3DVH analysis

3D dose verification was performed, using the 3DVH software. The latter reconstructs in 3D the measured dose distribution allowing the comparison with the respective TPS‐calculated one. It enables the calculation of all DVHs of the structures of interest contoured for the treatment plan from the measurements which were recorded with ArcCHECK, through the “Planned Dose Perturbation,” PDPTM algorithm.

The operation of the AC‐PDP algorithm is explained in detail in the work of Zhen et al.^10^ In short, the PDP algorithm employs a process of “perturbation” to address dose differences in the distribution of TPS‐calculated plans, as identified through analysis of the measured dose distribution from ArcCHECK. This involves reconstructing the distribution of doses based on the identified discrepancies, resulting in a corrected dose map that accounts for the cold and hot spots. The software uses the dose differences found between the ArcCHECK measurement and the TPS dose calculation for each beam and projects those differences back into the TPS 3D dose calculation to obtain an estimate of the actual delivered 3D dose distribution. This process is performed for each voxel of the plan.

#### 2.3.1 Dosimetric data collection by 3DVH

In this study, 3D %GP were calculated for each treatment plan (prostate/H&N), focusing on PTVs and selected OARs. For the 3DVH analysis the same acceptance criterion was used as in the 2D analysis, that is, 3%/2  mm, low‐dose cutoff threshold 10% of the maximum dose and global normalization.

### 2.4 Tolerance limits (TL) and action limits (AL) calculation

The TL and AL for both 2D %GP and 3D %GP were calculated across the entire irradiated area, including the OARs and the PTV. The determination of these limits was based on the analysis of 30 treatment plans for each anatomical site, encompassing a range of plan complexity.

In accordance with TG‐218,^6^ TL, and AL were calculated with the following equations (Equations (1) and (2)):

(1) $$\mathrm{TL}=\bar{x}\;-2.660\times \;\frac{1}{n-1}\;\sum_{i=2}^{n}\left|x_{i}-x_{i-1}\right|$$

and

(2) $$\mathrm{AL}=100\%-\frac{\beta \;\times \sqrt{\left(\sigma^{2}+{\left(\bar{x}-T\right)}^{2}\right)}}{2}$$

where $\sigma^{2}$ and $\bar{x}$ are the variance and mean value of the measurements used for the calculation of the limits, *x_i_* = the result of each measurement, n is the number of measurements, *T* = 100% (the target value of the measurements), and constant 𝛽 = 6.0.^25^

### 2.5 Statistical analysis

Statistical analysis was performed using the Pearson *r* linear coefficient and the Spearman coefficient for datasets exhibiting non‐symmetrical distributions. If the coefficient was less than ± 0.10 there was no correlation; values between ± (0.10–0.40) imply a weak correlation and values between ± (0.40‐0.70) imply a moderate correlation. Values greater than ± 0.70 show strong correlation. Statistical significance was determined by a *p*‐value derived from the coefficient correlation (*r*) being less than 0.05. Pearson or Spearman coefficients were used for the correlation analysis of the following parameters: plan 2D %GP (total area) versus plan 3D %GP (total area), 2D %GP versus 3D %GP (individual structures). In addition, box‐plots were utilized to identify the outliers of 3D %GP H&N PTV. After removing the outliers, statistical analysis was performed again.

## 3 RESULTS

### 3.1 2D %GP and 3D %GP

The results for 2D %GP and 3D %GP are presented in Table 1. The average 2D %GP for H&N cases was 96.4% and for prostate cases was 96.9%, whereas for 3DVH analysis it was 97.9% for H&N cases and 98.7% for prostate cases.

**TABLE 1:** Descriptive characteristics of %GP in the H&N and prostate areas.

Anatomical site	Parameter	Structure	*N*	Range (%)	$\bar{\%\mathrm{GP}}$ ± SD%
H&N	2D %GP	Plan	67	84.8–100	96.4 ± 3.2
	3D %GP	Plan	67	83.2–100	97.9 ± 3.1
		PTV	67	53.9–100	92.7 ± 9.9
		Brainstem	45	75.0–100	98.6 ± 5.1
		Mandible	54	70.3–100	97.7 ± 5.7
		Oral cavity	55	62.7–100	96.5 ± 7.5
		Left parotid	50	93.5–100	99.5 ± 1.1
		Right parotid	51	85.6–100	98.4 ± 3.1
		Spinal cord	63	62.2–100	97.0 ± 7.6
		Chiasm	11	68.9–100	93.1 ± 11.4
		Left optic nerve	11	74.2–100	95.5 ± 9.0
		Right optic nerve	11	75.7–100	94.4 ± 9.0
Prostate	2D %GP	Plan	69	91.0–100	96.9 ± 2.6
	3D %GP	Plan	69	88.2–100	98.7 ± 1.6
		PTV	69	71.7–100	94.6 ± 10.0
		Right femur	69	92.1–100	99.7 ± 1.1
		Left femur	69	93.1–100	99.8 ± 0.9
		Rectum	69	48.5–100	90.5 ± 8.7
		Bladder	69	64.0–100	96.9 ± 5.7
		Small bowel	38	97.1–100	99.7 ± 0.6

Abbreviations: %GP, Gamma passing rate; H&N, head and neck; PTV, planning target volume; SD, standard deviation.

### 3.2 Statistical analysis of %GP

The results from the statistical analysis of the %GPs are shown in Table 2. For H&N plans there was a strong (*r* > 0.70) correlation between plan 3D %GP and individual 3D %GP of PTV, brainstem, mandible, oral cavity, chiasm, and left and right optic nerve.

**TABLE 2:** Correlation indices between 3D and 2D %GP (plan) with the individual 3D %GP for the anatomical sites of H&N and prostate.

			3D %GP		2D %GP	
Anatomical site	%GP	Structure	*r*	*p*	*r*	*p*
H&N	2D %GP	Plan	0.45	<0.01	–	–
	3D %GP	PTV	0.85	<0.01	0.39	<0.01
		Brainstem	0.81	<0.01	0.48	<0.01
		Mandible	0.81	<0.01	0.33	0.02
		Oral cavity	0.83	<0.01	0.26	0.059
		Left parotid	0.45	<0.01	0.23	0.106
		Right parotid	0.67	<0.01	0.40	0.003
		Spinal cord	0.69	<0.01	0.51	<0.01
		Chiasm	0.77	<0.01	0.72	0.012
		Left optic nerve	0.74	<0.01	0.45	0.17
		Right optic nerve	0.71	0.01	0.38	0.251
Prostate	2D %GP	Plan	0.59	<0.01	–	–
	3D %GP	PTV	0.93	<0.01	0.17	0.154
		Right femur	0.32	<0.01	0.06	0.630
		Left femur	0.86	<0.01	0.01	0.941
		Rectum	0.69	<0.01	0.47	<0.01
		Bladder	0.55	<0.01	0.03	0.842
		Small bowel	0.59	<0.01	0.18	0.483

Abbreviations: %GP, Gamma passing rate; H&N, head and neck; PTV, planning target volume.

There was a moderate ± (0.40–0.70) correlation between the plan 3D %GP and the individual 3D %GP of parotids and spinal cord, as well as with the plan 2D %GP. However, there was a weak correlation (*r* < 0.40) between plan 2D %GP and the individual 3D %GP of the right optic nerve. 2D %GP seems to have a strong correlation with the individual 3D %GP of chiasm, and a moderate correlation with the following 3D %GP of OARs: brainstem, right parotid and spinal cord, and left optic nerve. It should be noted that the 2D %GP had a weak correlation with 3D %GP of PTV.

Regarding %GP of prostate treatment plans there was a strong correlation between the plan 3D %GP and the 3D %GP of PTV, as well as with the following 3D %GP of the following OARs: left femur, rectum, and bladder. A moderate correlation appeared between plan 3D %GP and the 3D %GP of small bowel, as well as with the plan 2D %GP, while a weak correlation between plan 3D %GP and the 3D %GP of right femur. The 2D %GP had a weak correlation with all the individual 3D %GP of OARs and PTV, except for the rectum (moderate correlation).

Overall, it is apparent that the correlations between 3D %GP were stronger than those between 2D %GP with PTV 3D %GP and OAR 3D %GP.

### 3.3 Tolerance limits (TL) and action limits (AL)

Tables 3 and 4 show the TL and AL calculated from 30 H&N and prostate plans, (mean value and range) for each anatomical structure. Regarding 2D %GP, the TL were narrower by 1.6% and 1.9% for the H&N and prostate cases respectively, compared to the TG‐218 protocol limits. The respective AL had a wider range by 5.6% and 4.2%. Regarding the plan 3D %GP, the TL were at 97.5% for the H&N cases and 98.8% for the prostate cases. Τhe corresponding AL were 87.7% and 94.8%.

**TABLE 3:** Tolerance and action limits (TL and AL) calculated from the selected plans, their mean, and range of values for each anatomical structure in the H&N area.

H&N structure	*N*	Range (%)	$\bar{\%\mathrm{GP}}$± SD%	TL	AL
2D plan	30	84.8–100	96.5 ± 3.9	96.6	84.4
3D plan	30	86.4–100	97.4 ± 3.2	97.5	87.7
PTV	30	53.9–100	89.2 ± 12.6	89.2	49.6
Brainstem	30	75.0–100	99.1 ± 4.6	99.1	86.0
Mandible	30	70.3–100	97.4 ± 6.2	97.4	79.9
Oral cavity	30	81.7–100	96.5 ± 5.7	96.6	80.0
Left parotid	30	93.5–100	99.5 ± 1.4	99.5	95.7
Right parotid	30	89.6–100	98.6 ± 2.7	98.6	90.8
Spinal cord	30	64.1–100	95.4 ± 8.3	95.4	71.6

Abbreviations: %GP, Gamma passing rate; AL, action limits; H&N, head and neck; PTV, planning target volume; SD, standard deviation; TL, tolerance limits.

**TABLE 4:** Tolerance and action limits (TL and AL) calculated from the selected plans, their mean, and range of values for each anatomical structure in the prostate area.

Prostate structure	*N*	Range (%)	$\bar{\%\mathrm{GP}}$± SD%	TL	AL
2D plan	30	95.5–100	98.7 ± 1.3	96.9	86.8
3D plan	30	96.2–100	98.7 ± 1.2	98.8	94.8
PTV	30	80.7–100	96.2 ± 5.6	96.1	79.6
Right femur	30	98.0–100	99.9 ± 0.4	99.9	98.6
Left femur	30	96.9–100	99.8 ± 0.6	99.8	98.1
Rectum	30	68.6–100	90.6 ± 8.2	93.4	62.3
Bladder	30	64.0–100	96.9 ± 6.8	96.9	77.8
Small bowel	30	97.1–100	99.7 ± 0.7	99.6	97.8

Abbreviations: %GP, Gamma passing rate; AL, action limits; PTV, planning target volume; SD, standard deviation; TL, tolerance limits.

Tables 5 and 6 categorize the plans according to the %GP of the treatment plan and individual structure(s) for the institutional and the TG‐218 TL and AL. They fell into three categories, namely, acceptable, under investigation or unacceptable, depending on whether they agreed with the calculated values of the present study and of the TG‐218 protocol limits. However, the latter (TL and AL) are defined only for the plan 2D %GP in the TG‐218 protocol. The results in plan %GP percentages between the two anatomical sites (H&N – prostate) appear to be similar.

**TABLE 5:** Classification of %GPs into acceptable, under investigation and unacceptable according to the TL and ΑL found in this study and in relation to the respective TG‐218 protocol for the H&N area.

H&N	Institutional limits			TG‐218 limits		
Structure	%GP ≥ TL	AL ≤%GP < TL	%GP < AL	%GP ≥ 95	90 ≤%GP < 95	%GP < 90
2D plan	37	30	0	49	15	3
3D plan	47	18	2	60	5	2
PTV	45	22	0	41	8	18
Brainstem	40	5	0	43	0	2
Mandible	46	10	0	50	3	3
Oral cavity	42	11	2	44	4	7
Left parotid	38	11	1	49	1	0
Right parotid	40	9	2	44	4	2
Spinal cord	52	9	2	53	4	6
SUM	387	125	9	433	44	43
Rate (%)	74.3	24.0	1.7	83.3	8.5	8.3

Abbreviations: %GP, Gamma passing rate; AL, action limits; H&N, head and neck; PTV, planning target volume; SD, standard deviation; TL, tolerance limits.

**TABLE 6:** Classification of %GPs into acceptable, under investigation, and unacceptable according to the TL and ΑL found in this study and in relation to the respective TG‐218 protocol for the prostate area.

Prostate	Institutional limits			TG‐218 limits		
Structure	%GP ≥ TL	AL ≤% GP < TL	%GP < AL	%GP ≥ 95	90 ≤%GP < 95	%GP < 90
2D plan	43	26	0	49	9	11
3D plan	43	25	1	68	1	0
PTV	43	23	3	53	8	8
Right femur	64	4	1	68	1	0
Left femur	63	3	3	68	1	0
Rectum	44	24	1	26	18	25
Bladder	54	13	2	59	7	3
Small bowel	31	6	1	69	0	0
SUM	385	124	12	460	45	47
Rate (%)	73.9	23.8	2.3	83.3	8.2	8.5

Abbreviations: %GP, Gamma passing rate; AL, action limits; PTV, planning target volume; SD, standard deviation; TL, tolerance limits.

**FIGURE 1:**
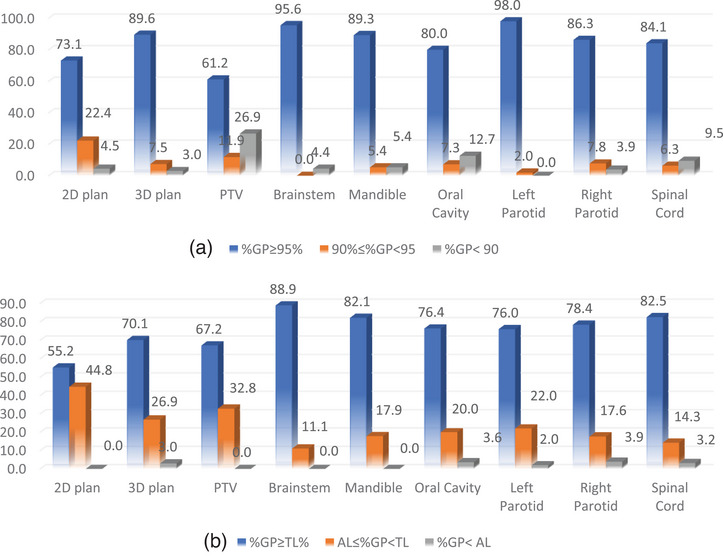
(a) Frequency rates (%) of acceptable plans, plans under investigation, and unacceptable plans for each structure separately and plan %GPs for H&N area with respect to the limits set by this study and (b) by TG‐218. H&N, head and neck.

**FIGURE 2:**
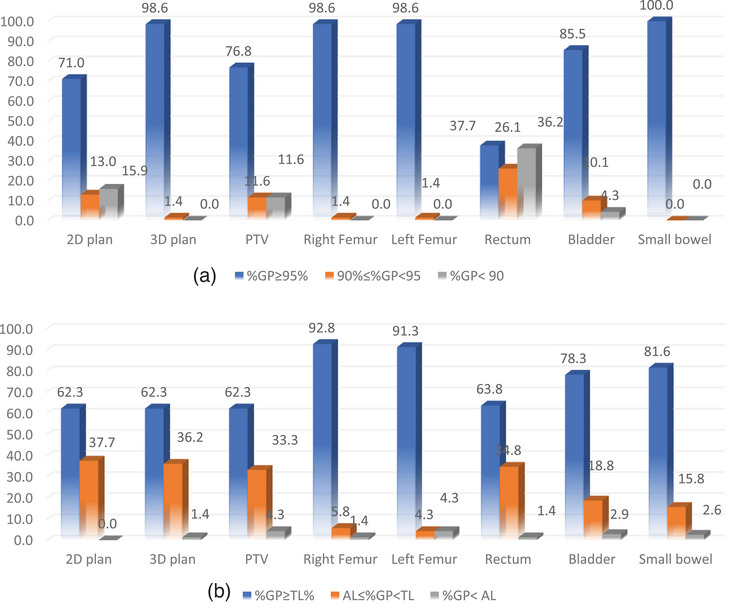
(a) Frequency rates (%) of acceptable plans, plans under investigation, and unacceptable plans for each structure separately and plan %GPs for prostate area with respect to the limits set in this study and (b) in TG‐218. %GP, Gamma passing rate.

In Figures 1 and 2, the treatment plans are classified based on both plan and individual %GP regarding the tolerance limits (TL) and action limits (AL) established by the institution and TG‐218 guidelines for head and neck (H&N) and prostate patients, respectively. The figures depict the percentage frequency rates of plans falling into the categories of acceptable, under consideration, and unacceptable %GPs. This classification is presented for both anatomical sites, comparing the limits defined in this study with those recommended by TG‐218.

The percentage of acceptable plans with respect to the limits set by this study for the H&N cases ranged from 55.2% to 88.9%. The corresponding percentage for the plans under consideration ranged from 11.1% to 44.8%, while for the unacceptable plans ranged from 0.0% to 3.6%. (see Figure 1a) The percentage of acceptable plans with respect to the limits published by TG‐218 for the H&N cases range from 73.1% to 98.0%, for the plans under consideration range from 0.0% to 11.9%, while for the unacceptable plans range from 0.0% to 12.7%. (see Figure 1b). It was observed that plan 3D %GP percentages for acceptable plans were higher than plan 2D %GP by approximately 15%

Regarding the prostate cases, the acceptable plans with respect to the limits set by this study for the prostate cases had a percentage of 62.3% to 92.8%. The corresponding plans under consideration had a percentage of 4.3% to 37.7%. Unacceptable plans had a percentage of 0.0% to 4.3%. (see Figure 2a).

Acceptable plans in terms of the limits set in TG‐218 for the H&N area had a percentage of 37.7% to 100%. Plans under consideration had a percentage of 0.0% to 26.1% and unacceptable plans had a percentage (%) of 0.0% to 15.9%. (see Figure 2b).

## 4 DISCUSSION

This study focused on the investigation of a PSQA method used complimentary to the currently used 2D %GP. While 2D %GP has proven to be a useful QA tool, it lacks anatomical information about the locations where discrepancies occur. Consequently, the medical physicist faces challenges in discerning the extent of divergence between the two dose distributions. Το overpass the limitations of the 2D planar dose distribution, a 3D QA method based on actual patient anatomy was tested. For that purpose, the 3DVH software was utilized and the correlation between the 2D and 3D %GPs was investigated in order to develop an optimized gamma analysis approach. Furthermore, this study facilitated the assessment and establishment of institution‐specific TL and AL of 2D %GP and 3D %GP for both treatment plan and individual structures.

Following a retrospective analysis of 2D %GP, and plan and individual 3D %GP, it was observed that 3D %GP seem to exhibit higher values than the 2D %GP. Tables 1 and 2 show that both the 3D %GP (plan and individual) and 2D %GP are systematically lower for H&N cases than for prostate ones. The lower %GP found for the H&N cases can attributed to the increased complexity (increased dose distribution modulation) of the H&N plans, as compared to prostate plans. Moreover, the %GP ranges of prostate exhibited a shorter range of values. The mean treatment plan 2D %GP for H&N cases was 96.4% while for prostate cases was 96.9%. The treatment plan 3D %GP was 97.9% for H&N and 98.7% for prostate cases. These results appear to be in agreement with similar studies. Ιn such a study, Song et al.^18^ used a 2D diode detector array (MapCHECK 2) for 20 IMRT patients, 10 with H&N cancer, and 10 with prostate cancer), and studied the 2D %GP, as well as 3D %GP using 3DVH software. When the 3%/3  mm acceptance criterion was applied, the plan %GP was 96.2% and 98.3% for H&N and prostate cases respectively in 2D analysis. Performing 3DVH analysis, for H&N and prostate cases, plan 3D %GP was 97.9% and 99.4% respectively.

Through the correlations of the plan 2D and 3D %GP with the individual 3D %GP of the structures of interest, it was observed that the plan 3D %GP correlated stronger (0.32 ≤ *r* ≤ 0.93) with the individual 3D %GP of both PTV and OARs than the 2D %GP (0.01 ≤ *r* ≤ 0.72). Treatment plan 3D %GP correlated with individual PTV 3D %GP as follows: *r* = 0.85 for PTV Η&Ν and *r* = 0.93 for PTV prostate while plan 2D %GP were correlated with corresponding individual PTV 3D %GP as follows: *r* = 0.39 for PTV Η&Ν και *r* = 0.17 for PTV prostate. Thus, it appears that the 3D gamma index resulted in a higher correlation with the clinical plan than the corresponding 2D analysis.

An interesting fact (Table 1) is that the lowest average 3D %GP for the prostate cases is exhibited by the rectum (90.5) and for the H&N cases by the chiasm (93.1). The reason(s) behind this are still under investigation and fall beyond the scope of this work; however they could be of different cause; the rectum is at a steep dose gradient region, that overlaps with the PTV, resulting in possibly more failed points; similarly, the chiasm could be found near a steep dose gradient region and/or it could be that its low passing rate could be attributed to its small volume, as even a few points could heavily affect its 3D %GP. In addition, through the analysis of the correlation between the plan 3D %GP and structure 3D %GP (Table 2) for the prostate plans, the correlation of the right and left femoral heads with respect to the plan exhibits a notable difference (0.32 right femoral head, 0.86 left femoral head). In the case of H&N plans the parotids’ correlation exhibits a similar difference (0.67 right parotid, 0.45 left parotid). This is an interesting finding, as one would expect similar values for bilateral structures. However, this discrepancy is not systematic, as it is different in the two treatment sites. Sych difference could be related to the size, volume and extension of the PTV with respect to the aforementioned OARs. A larger number of plans could provide a more definitive answer to this finding. Even though further analysis is beyond the scope of the current work, such findings can demonstrate how dosimetric and clinically meaningful features can be extracted from such structure‐individual 3D %GP analysis.

To the best of our knowledge, correlations between plan 3D %GPs and individual (PTV and/or OAR) 3D %GPs have not been previously reported in the literature for H&N or prostate area. Previous studies were restricted to a comparison between 2D %GP and individual (PTV, and/or OAR) 3D %GP.^19^, ^20^

The correlation between plan 2D and plan 3D %GP was moderate, for H&N cases (*r* = 0.45), as well as prostate cases (*r* = 0.59). Song et al.^18^ studied the Pearson coefficients in the correlation between the individual 3D %GP of PTV and OARs with the 2D %GP and concluded that the correlation for each individual structure may be almost zero or slightly negative, although the correlation for the total calculated volume was strongly positive (*r* = 0.83). A similar analysis was performed by Zhang et al.^19^ who correlated 2D %GP with 3D %GP for all 154 prostate plans with different gamma evaluation criteria (2%/1, 3%/2, and 3%/3 mm). A significant correlation was found between the 2D and 3D %GPs for all gamma evaluation criteria tested, both for the whole body and the PTV region (*r* > 0.8 and *p* < 0.001). Similar results to those of Zhang et al. were reported by Wu et al.^26^ who found a statistically significant correlation between 3D and 2D plan %GPs in their study of two IMRT QA methods. The results of our study exhibit lower correlation values; this discrepancy could be attributed to the different irradiation technique (VMAT vs. IMRT), different dose verification system (3D vs. 2D array), or different clinical protocols overall.

Regarding the TL and AL, they may be different between institutions, as each clinic might use a different methodology for plan QA. Although the institutional TL and AL for 2D %GP were estimated by Stasinou et al.,^21^ they were also investigated in the present study, without the intention of comparison.

A major outcome of this study was the calculation of the TL and AL from selected treatment plans for plan 3D %GP and individual 3D %GP of each structure using statistical methods following TG‐218 rationale.

In terms of 2D %GP, TL are 96.6% and 96.9% for H&N and prostate cases, respectively, with corresponding AL are 84.4% and 86.8%. In the study by Stasinou et al.,^21^ TL and AL were exclusively explored for 2D %GP. 2D %GP TL for H&N cases it was 92.2%, while for prostate cases it was 93.8%. The corresponding AL were 88.5% and 90.5% for H&N and prostate cases, respectively. In a similar study, Deng et al.^27^ conducted a study in which the 2D %GP TL and AL limits of VMAT and IMRT plans were calculated for the head, chest, and pelvis areas using SUNcheck. As for the 2D, the %GP TL for H&N cases was 94.65% and AL was 93.98%.

In the current study regarding the TL for the treatment plan 3D %GP, they were found to be 97.5% and 98.8% for H&N and prostate cases, respectively, and the corresponding AL were calculated at 87.7% and 94.8%. The lower limits found for the H&N cases are likely attributed to the increased complexity of the H&N plans, which degrades the %GP. As for the TL of the individual 3D %GP, they appear to be higher than the TL established by the TG‐218 protocol for the 2D %GP, except for the PTV in H&N cases.

Boxplots were utilized to identify the outliers of 3D %GP H&N PTV. After removing the outliers, statistical analysis was performed again. This led to an increase in TL of Η&Ν PTV from 89.2 to 93.6. The 3D %GP AL of each structure in the H&N cases was found to be lower than 90%, except for the parotid structures. It is also observed that the low ΑL of Η&Ν PTV, when outliers were removed, increased from 49.6 to 72.9.

The 3D %GP AL of each structure in the prostate cases was found to be higher than 90%, except for PTV, rectum, and bladder. The lower TL and AL limits observed in the H&N PTV area compared to prostate PTV could be attributed to the inherently more complex dose distribution in H&N plans, as compared to the relatively simpler dose distribution in prostate cases.

Concerning the TL and AL of OARs, they were found higher than the corresponding PTV TL and AL for both anatomical areas, except for TL and AL Rectum and AL Bladder. A similar analysis of TL and AL of 3D %GP has not been reported in the literature. It is essential to highlight that a high plan %GP in either 3D or 2D analysis, does not consistently correlate with a high individual 3D %GP (such as in the case of PTV‐ 3D %GP, rectum‐ 3D %GP). Similarly, a low overall %GP does not necessarily indicate a low %GP in a specific organ volume. To accurately depict the results for each structure, a comprehensive 3D analysis of each individual structure is essential and beyond the scope of the current work.

From the analysis of Tables 3 and 4, it can be concluded that the AL calculated for the 2D %GP with the 3%/2 mm criterion were lower than those proposed by the TG‐218, while the TL were higher.

Furthermore, it was observed that the percentage of plan 3D %GP for acceptable plans, in accordance with TG‐218 limits, was approximately 17% lower for H&N and 27% lower for prostate compared to 2D %GP. Additionally, with respect to the institutional limits the percentages of acceptable plans, as well as plans under consideration and unacceptable ones show significant differences.

A limiting factor of this study stems from the use of sequential boosts, resulting in each patient having multiple treatment plans. As a result, a comprehensive analysis of the total dose received by each structure in the region of interest was not feasible. Additionally, this study encompassed H&N plans of various treatment sites, such as larynx, oropharynx and nasopharynx; no further sub‐categorization was considered. Finally, the absence of established guidelines for TL and AL for 3D %GP (treatment plan and/or individual), prevents comparative analysis and the derivation of more qualitative inferences.

## 5 CONCLUSIONS

The 3D %GP analysis of VMAT PSQA demonstrated more robust correlations with the individual 3D %GP values of PTV and OARs compared to 2D %GP. This underscores the inadequacy of relying solely on 2D %GP analysis for PSQA. The findings advocate for the establishment of institutional tolerance (TL) and action (AL) limits, specifically tailored for PTV and OARs. The study emphasizes the increased value of 3D PSQA methods, providing enhanced insights and assurance regarding the clinical plans. Consequently, there is a need for medical physicists to develop an optimized PSQA method that strikes a balance between ensuring safety and maintaining time efficiency, particularly in departments with heavy workloads. This imperative arises from the study's compelling evidence that a more comprehensive and sophisticated approach to PSQA is essential for delivering high‐quality and safe clinical treatments.

## AUTHOR CONTRIBUTIONS

*Conception of the project*: Kalliopi Platoni, George Patatoukas, and Christos Zarros. *Collection and analysis of data for the work*: Christos Zarros. *Investigation of data*: Christos Zarros, George Patatoukas, Nikos Kollaros, and Marina Chalkia. *Review and editing of the work*: Kalliopi Platoni, George Patatoukas, Marina Chalkia, Nikolaos Kollaros, Andromachi Kougioumtzopoulou, and Vasilios Kouloulias. *Final approval of the version to be published*: Kalliopi Platoni. All authors have read the published version of the manuscript and agreed on all aspects of the work.

## CONFLICT OF INTEREST STATEMENT

The authors declare no conflicts of interest.

## Data Availability

The authors will share data upon request to the corresponding author.
